# Genetic Expression between Ageing and Exercise: Secreted Protein Acidic and Rich in Cysteine as a Potential “Exercise Substitute” Antiageing Therapy

**DOI:** 10.3390/genes13060950

**Published:** 2022-05-26

**Authors:** Abdelaziz Ghanemi, Mayumi Yoshioka, Jonny St-Amand

**Affiliations:** 1 Functional Genomics Laboratory, Endocrinology and Nephrology Axis, CHU de Québec-Université Laval Research Center, Quebec, QC G1V 4G2, Canada; abdelaziz.ghanemi@crchudequebec.ulaval.ca (A.G.); mayumi.yoshioka@crchudequebec.ulaval.ca (M.Y.); 2 Department of Molecular Medicine, Faculty of Medicine, Laval University, Quebec, QC G1V 0A6, Canada

**Keywords:** secreted protein acidic and rich in cysteine, ageing, exercise, antiageing

## Abstract

Ageing is the effect of time on biological entities. It represents a risk factor for a variety of diseases and health disorders; thus, therapeutic options are required to tackle ageing issues. Modern geriatric medicine prescribes exercise to counteract ageing effects. This work presents secreted protein acidic and rich in cysteine (SPARC) as a potential antiageing therapy. Indeed, SPARC declines with ageing, exercise induces SPARC, and SPARC overexpression in mice mimics exercise. Thus, we hypothesize that SPARC is an exercise-induced factor that is beyond—at least part of—the antiageing effects induced by exercise. This could become a potential antiageing therapy for the elderly that counteracts ageing by mimicking the effects of exercise without needing to perform exercise. This is of particular importance because ageing usually reduces mobility and age-related diseases can reduce the ability to perform the required physical activity. On the other hand, the possibilities of mimicking exercise benefits via SPARC are not limited to ageing, and can be applied in various contexts in which exercise cannot be performed because of physical disabilities, health disorders, or limited mobility.

Ageing is defined as the biological decline of diverse functions and processes within cells, tissues, and organisms over time [1,2]. Biological ageing can also be defined as the cellular and tissue changes that develop through one’s lifespan. These changes include metabolic decline [3], skeletal muscle mass loss [4], adipose tissue dysfunction [5,6], cognitive decline [7], and immunosenescence [8]. Ageing involves molecular and cellular changes such as epigenetic modifications, inflammation, and impaired regeneration [2]. Delaying ageing has been the focus of humans for a long time, with ancient philosophers/civilizations describing the fountain of youth [9]. Millennia later, the development of healthcare systems has led to ageing societies [10]. Ageing is an important risk factor for various diseases and health problems. Thus, biomedical research is focused on how to tackle ageing and diverse studies have pointed out factors that could contribute to either slowing down ageing or accelerating it. Both exercise [11,12] and calorie restriction [13] are among the most well-known approaches to counteracting the effects of ageing. More specifically, the diverse benefits of exercise [14,15,16,17,18,19] are the reason it is prescribed to the elderly in order to counteract/limit the metabolic and functional decline associated with ageing. Therefore, we suggest the existence of molecular patterns shared between ageing and exercise, as two physiological changes, that can explain the antiageing effect of exercise. Understanding the mechanistic links between exercise and antiageing effects at molecular and cellular levels will allow us to deepen our knowledge towards developing and optimizing antiageing therapies.

Thus, there are potentially molecular pathways beyond the antiageing effect of exercise. Within this context, here, we specifically focus on secreted protein acidic and rich in cysteine (SPARC). *SPARC/Sparc* has been identified as a gene with an expression level that changes with both exercise and ageing. Interestingly, these changes take place in opposite directions. Indeed, while exercise (as well as the in vivo model of exercise) increases the *SPARC*/*Sparc* expression [20,21,22], this gene expression decreases with ageing [21]. Such expression patterns indicate that SPARC represents a key molecular pathway in both exercise and ageing, and explain, at least in part, both the ageing process and the antiageing effects of exercise. In addition, the effects of exercise on skeletal muscle counteract those of ageing [23]. Not only has *SPARC* been characterized as an exercise-induced gene, but *Sparc* KO in mice or SPARC inhibition in the cell culture leads to an ageing-like phenotype [24,25]. Moreover, SPARC overexpression mimics exercise-induced changes [24]. Therefore, it seems that a decrease in *SPARC* expression might contribute to the ageing process, while an increase in *SPARC* expression could be involved in the exercise-induced changes. Although more evidence is still required, we focused on SPARC because we have shown that it is extremely upregulated by exercise (aerobic exercise rather than resistance training [26,27]) compared with other exercise-induced genes [28], in addition to being downregulated with ageing [21]. The measure of SPARC/*SPARC*/*Sparc* expression has also been suggested as a molecular physiological and pathological biomarker [29], as well as a molecular tool to optimize personalized medicine based on exercise prescription [30].

We previously suggested that the antiageing effect of exercise might be mediated by the exercise-induced increase in SPARC expression, which reverses/counteracts the ageing-associated decline in SPARC/*Sparc* expression [24]. This is supported by a study suggesting that exercise-induced muscle phenotype changes are SPARC-dependent [31]. The association between the ageing phenotype and SPARC decline is further supported by the fact that animal models of *Sparc* KO exhibit ageing-like phenotypes, including accelerated degeneration [32,33], osteopenia [34], early onset of cataractogenesis [35,36], lack of immune response to lipopolysaccharides [37], and decreased bone formation [38]. Furthermore, the involvement of SPARC in exercise-induced antiageing effects is confirmed by SPARC overexpression in mice [24] or the addition of SPARC to the muscle cell cultures [25], which also mimics exercise in terms of metabolism and muscle properties. Therefore, SPARC expression levels could be an indicator of whether the phenotype would be for ageing (low SPARC expression) or rather an exercise-induced (antiageing) phenotype (high SPARC expression).

On the one hand, the similarities between *SPARC* properties and exercise-induced effects and the *SPARC*-induced effects indicate that *SPARC* acts towards counteracting ageing; on the other hand, they represent elements that present *SPARC* as a molecule that can both mimic exercise and counteract ageing. Indeed, SPARC has been shown to have diverse properties, such as anti-inflammatory [39], anticancer [40], and regenerative properties [41]. SPARC is also involved in metabolism [42,43] and obesity [44], among others, all of which are properties that would be beneficial against ageing. Thus, SPARC would be a selective target towards a potential antiageing therapy. This could be achieved either by injecting SPARC; inducing *SPARC* expression (gene therapy); or, as a more specific therapy, stimulating selected SPARC-induced pathways. Such an approach would generate antiageing effects, including those induced by exercise (Figure 1). The result would be an antiageing therapy for the elderly that counteracts ageing by mimicking the effects of exercise without the need to do exercise. This is of particular importance, because ageing usually reduces mobility and age-related diseases could also reduce the ability to perform the required physical activity.

These *SPARC*-related properties illustrate how genetics might contribute to developing and optimizing antiageing therapies. Functional genomics studies the changes in gene expression under various conditions, including diet [45,46], ageing [47,48], and exercise [49]. The aim of our hypothesis, presented herein, is to target gene(s) that are both overexpressed during exercise and at the same time downregulated with ageing. This expression pattern suggests that such gene(s) are involved in both ageing and exercise (antiageing).

Based on the fact that SPARC declines with ageing and that exercise induces SPARC, we hypothesized that SPARC is an exercise-induced antiageing factor, after we showed that SPARC overexpression mimics the effects of exercise. The same logic could be carefully applied to diet. Indeed, diets such as calorie restriction diets are prescribed to counteract the effects of ageing. Thus, studying the variations in gene expression induced by such diets and how the expressions of such genes change with ageing could identify novel targets. Pharmacological intervention on such targets would mimic the therapeutic outcome of calorie-restriction diets (antiageing). On the other hand, and although more evidence is required, the possibilities of mimicking exercise benefits via SPARC are not limited to ageing and could be applied in various contexts in which exercise cannot be performed because of physical disabilities, health disorders, or limited mobility.

## Figures and Tables

**Figure 1:**
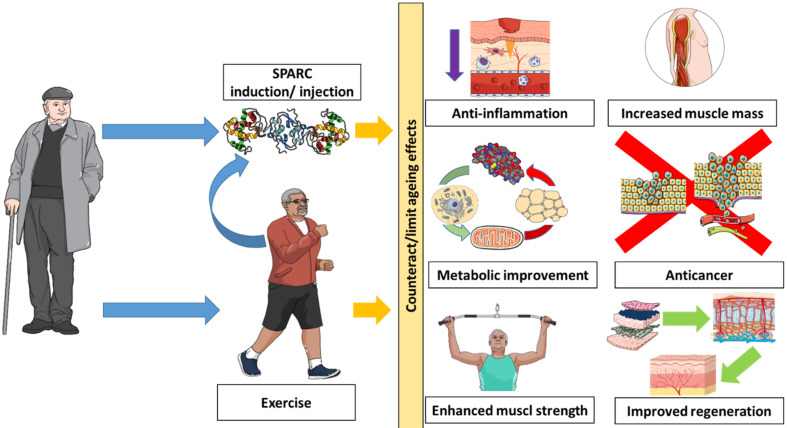
Secreted protein acidic and rich in cysteine (SPARC) as a potential antiageing “exercise substitute”. SPARC (which is induced by exercise) represents a potential therapy that can mimic exercise and produce antiageing effects. This is of particular importance because ageing usually reduces mobility and age-related diseases could also reduce the ability to perform the required physical activity.
